# Future Earth Health Knowledge-Action Network

**DOI:** 10.1186/s40985-016-0039-y

**Published:** 2016-11-16

**Authors:** Paul Shrivastava, Kari Raivio, Fumiko Kasuga, Joshua Tewksbury, Andy Haines, Peter Daszak

**Affiliations:** 1 Future Earth Secretariat, Montreal, Canada; 2 Future Earth Engagement Committee, Helsinki, Finland; 3 Future Earth Secretariat, Tokyo, Japan; 4 Future Earth Secretariat, Boulder, CO USA; 5 London School of Hygiene & Tropical Medicine, London, UK; 6 EcoHealth Alliance, New York, NY USA

**Keywords:** Future Earth, Human health, Planetary health, Sustainability research, Climate change, Knowledge-Action Networks

## Abstract

Future Earth is an international research platform providing the knowledge and support to accelerate our transformations to a sustainable world. Future Earth 2025 Vision identified eight key focal challenges, and challenge #6 is to “Improve human health by elucidating, and finding responses to, the complex interactions amongst environmental change, pollution, pathogens, disease vectors, ecosystem services, and people’s livelihoods, nutrition and well-being.” Several studies, including the Rockefeller Foundation/Lancet Planetary Health Commission Report of 2015, the World Health Organization/Convention on Biological Diversity report and those by oneHEALTH (former ecoHEALTH), have been conducted over the last 30 years.

Knowledge-Action Networks (KANs) are the frameworks to apply Future Earth principles of research to related activities that respond to societal challenges. Future Earth Health Knowledge-Action Network will connect health researchers with other natural and social scientists, health and environmental policy professionals and leaders in government, the private sector and civil society to provide research-based solutions based on better, integrated understanding of the complex interactions between a changing global environment and human health. It will build regional capacity to enhance resilience, protect the environment and avert serious threats to health and will also contribute to achieving Sustainable Development Goals. In addition to the initial partners, Future Earth Health Knowledge-Action Network will further nourish collaboration with other on-going, leading research programmes outside Future Earth, by encouraging them in active participation.

## Background

Future Earth is an international research platform providing the knowledge and support to accelerate transformations to a sustainable world. Future Earth brings together existing programmes on global environmental change, as well as experts from all disciplines, including natural and social sciences, engineering, the humanities, health, law and business, with a range of stakeholders including policymakers, to develop transdisciplinary solutions to sustainability challenges. Our Vision 2025 identifies eight key focal challenges as:

1. Deliver water, energy and food for all, and manage the synergies and trade-offs amongst them, by understanding how these interactions are shaped by environmental, economic, social and political changes
2. Decarbonise socio-economic systems to stabilise the climate by promoting the technological, economic, social, political and behavioural changes enabling transformations, while building knowledge about the impacts of climate change, and adaptation responses for people and ecosystems
3. Safeguard the terrestrial, freshwater and marine natural assets underpinning human well-being by understanding relationships between biodiversity, ecosystem functioning and services and developing effective valuation and governance approaches
4. Build healthy, resilient and productive cities by identifying and shaping innovations that combine better urban environments and lives with declining resource footprints and provide efficient services and infrastructures that are robust to disasters
5. Promote sustainable rural futures to feed rising and more affluent populations amidst changes in biodiversity, resources and climate by analysing alternative land uses, food systems and ecosystem options and identifying institutional and governance needs
6. Improve human health by elucidating, and finding responses to, the complex interactions amongst environmental change, pollution, pathogens, disease vectors, ecosystem services and people’s livelihoods, nutrition and well-being
7. Encourage sustainable consumption and production patterns by understanding the social and environmental impacts of consumption of all resources, opportunities for decoupling resource use from growth in well-being and options for sustainable development pathways and related changes in human behaviour
8. Increase social resilience to future threats by building adaptive governance systems, developing early warning of global and connected thresholds and risks and testing effective, accountable and transparent institutions that promote transformations to sustainability

To coordinate development of knowledge and action on these challenges, Future Earth is developing new inter- and transdisciplinary approaches on three themes of Dynamic Planet, Global Sustainable Development and Transformations towards Sustainability. The platforms for collaboration developed are called Knowledge-Action Networks (KANs).

## Main text

### Knowledge-Action Networks (KANs)

Knowledge-Action Networks constitute the framework for applying Future Earth approaches to research and related activities that respond to societal challenges. The main method of the Knowledge-Action Networks is facilitating high-quality actionable scientific knowledge through the integration of research and the involvement of societal partners, following the engagement guidelines of Future Earth. Knowledge-Action Networks build on the broad range and diverse specialist expertise represented in the large community of researchers and practitioners within the Future Earth structure, as well as endorsed and associated organisations, projects and individuals that want to join the Future Earth Open Network.

The objectives of the Knowledge-Action Networks are to:

- *Identify and respond to society’s needs* for scientific knowledge to successfully undertake the transformation to sustainability
- *Generate integrated knowledge* that is relevant to decision-makers
- *Develop and cultivate research* that is solution-driven, inter- and transdisciplinary
- *Add value to research* that is or has been carried out already

Participation in KANs is on a voluntary basis through members, projects or groups with the appropriate expertise and an interest in putting their expertise into the broader context of sustainability research addressed by Future Earth.

### Future Earth Health Knowledge-Action Network

In 2015, the Rockefeller Foundation-Lancet Commission on Planetary Health published its report: Safeguarding human health in the Anthropocene epoch [1]. It showed how the health of people is tightly linked to the health of the planet we inhabit and thus how adverse changes in the Earth’s natural ecosystems are a threat to human health. Advancing this concept requires wider understanding of the eco-social dimensions of health.

The Commission argued for urgent development of a new avenue of research inquiry—on planetary health. Attention to the human systems (economic, social and political) and Earth’s natural systems can improve health and well-being of all. The Future Earth Health KAN responds to this call, bringing health researchers together with other natural and social scientists, health and environmental policy experts and leaders in government, the private sector and civil society. The goal is to promote research-based solutions for better, integrated understanding of the complex interactions between a changing global environment (such as climate change, pollution, biodiversity loss, fishery declines and land use change), the loss of ecosystem services and the health of human beings (including livelihoods, nutrition and well-being). Long-term integrated observation systems to collect rigorous health, socio-economic and environmental data will be encouraged.

Another major focus will be to build regional capacity to integrate and act on planetary health knowledge to enhance resilience, protect the environment and avert serious threats to health. This work takes place in the context of the 17 Sustainable Development Goals approved by the United Nations Member States in 2015 [2], many of which deal directly or indirectly with health. Together with potential initial partners, including Future Earth’s oneHEALTH (former ecoHEALTH) project, the International Council for Science (ICSU) programme on Urban Health and Wellbeing, the World Health Organization and the United Nations University’s International Institute for Global Health, Future Earth Health KAN will further nourish collaboration with other on-going, leading research programmes.

## Conclusion

We anticipate that the development of this network will be a bottom up exercise lead by scientists, policy makers and stakeholders. We will be issuing a call for interest in late 2016 and early 2017 to identify voluntary participants. We encourage active participation from interested parties both in the scientific communities and in broader society.

## Abbreviations

KANs: Knowledge-Action Networks
